# Cardiovascular magnetic resonance of cardiac function and myocardial mass in preterm infants: a preliminary study of the impact of patent ductus arteriosus

**DOI:** 10.1186/s12968-014-0054-4

**Published:** 2014-07-23

**Authors:** Kathryn M Broadhouse, Anna E Finnemore, Anthony N Price, Giuliana Durighel, David J Cox, Anthony David Edwards, Joseph V Hajnal, Alan M Groves

**Affiliations:** 1 Imaging Sciences Department, MRC Clinical Sciences Centre, Imperial College, Hammersmith Hospital, London, UK; 2 The Centre for the Developing Brain, Division of Imaging Sciences & Biomedical Engineering, King’s College London, King’s Health Partners, St. Thomas’ Hospital, London SE1 7EH, UK

**Keywords:** Preterm, Cardiac function, Magnetic resonance, Patent ductus arteriosus

## Abstract

**Background:**

Many pathologies seen in the preterm population are associated with abnormal blood supply, yet robust evaluation of preterm cardiac function is scarce and consequently normative ranges in this population are limited. The aim of this study was to quantify and validate left ventricular dimension and function in preterm infants using cardiovascular magnetic resonance (CMR). An initial investigation of the impact of the common congenital defect patent ductus arteriosus (PDA) was then carried out.

**Methods:**

Steady State Free Procession short axis stacks were acquired. Normative ranges of left ventricular end diastolic volume (EDV), stroke volume (SV), left ventricular output (LVO), ejection fraction (EF), left ventricular (LV) mass, wall thickness and fractional thickening were determined in “healthy” (control) neonates. Left ventricular parameters were then investigated in PDA infants. Unpaired student t-tests compared the 2 groups. Multiple linear regression analysis assessed impact of shunt volume in PDA infants, p-value ≤ 0.05 being significant.

**Results:**

29 control infants median (range) corrected gestational age at scan 34^+6^(31^+1^-39^+3^) weeks were scanned. EDV, SV, LVO, LV mass normalized by weight and EF were shown to decrease with increasing corrected gestational age (cGA) in controls. In 16 PDA infants (cGA 30^+3^(27^+3^-36^+1^) weeks) left ventricular dimension and output were significantly increased, yet there was no significant difference in ejection fraction and fractional thickening between the two groups. A significant association between shunt volume and increased left ventricular mass correcting for postnatal age and corrected gestational age existed.

**Conclusion:**

CMR assessment of left ventricular function has been validated in neonates, providing more robust normative ranges of left ventricular dimension and function in this population. Initial investigation of PDA infants would suggest that function is relatively maintained.

## Background

Rates of premature births are increasing in absolute number and as a percentage of all births [1]. Many pathologies seen in this population are associated with abnormal or inadequate blood supply, yet robust assessment of the preterm cardiovascular system is limited. The transition from intra- to extra-uterine life requires abrupt changes in the cardiovascular system to maintain adequate systemic and pulmonary blood flow in the two extreme environments. The immature preterm cardiovascular system has undergone this transformation when it is not structurally equipped to do so, these circulatory changes are often delayed in this cohort [2],[3]. It is thought that this delay in circulatory adaption, the immature preterm cardiovascular system and the resulting haemodynamics correlate to the presence and development of the pathologies seen in this population [3]–[6].

**Figure 1:**
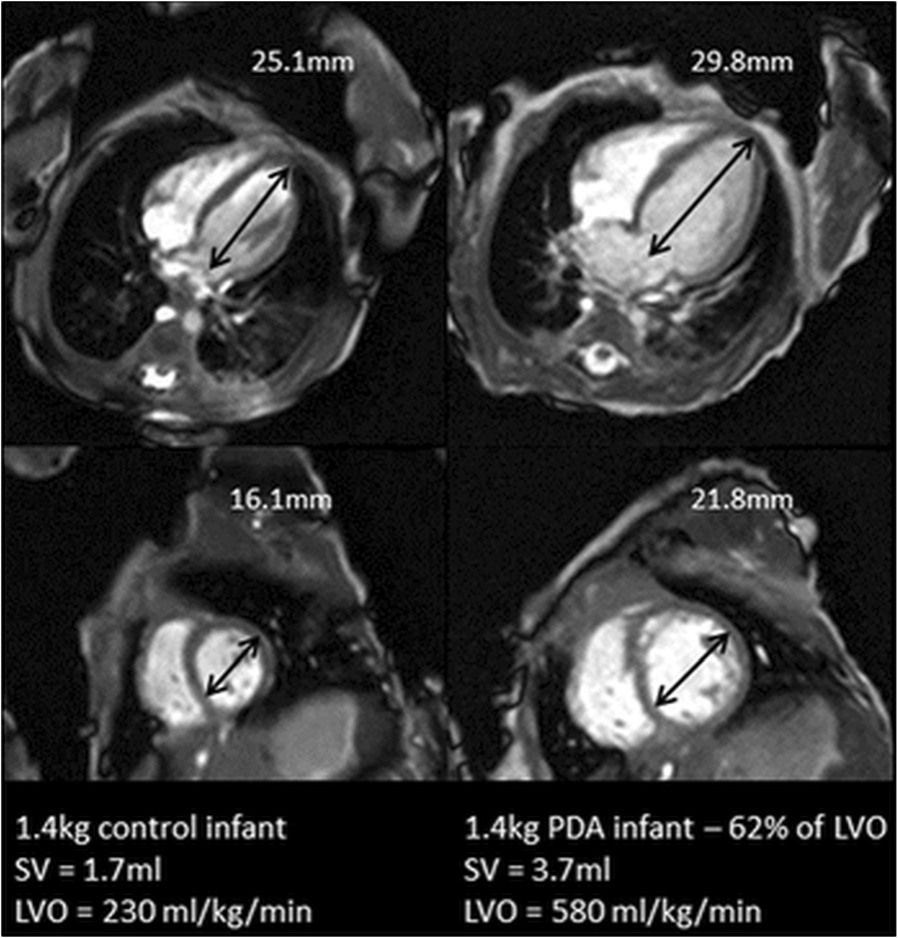
**Comparison of a 4 chamber and short axis view at end diastole in a 1.4 kg control infant (left) and 1.4 kg PDA (right) infant with a shunt volume of 62% of LVO.** Apex-base and mid cavity diameter measurements have been included for scale. Figure shows the apparent increase in left ventricular dimensions.

A common congenital defect in preterm infants clinically apparent in 60% of infants born less than 28 weeks gestation is a patent ductus arteriosus (PDA) [3]. It is common clinical belief that large ductal shunt volumes increase cardiac workload and are associated with congestive heart failure [2],[4],[7]; large shunt volumes are thought to lead to systemic hypo-perfusion and pulmonary hyper-perfusion due to cardiocirculatory dysfunction [8]–[10]. It has previously been shown that ductal shunt volume can range between 8-74% of left ventricular output (LVO) and that LVO is significantly increased in PDA infants [11],[12]. From observation in our previous study [12], infants with PDA appeared to have enlarged myocardium and larger ventricular cavity volumes than the “healthy” control preterm infants (Figure 1), yet the extent of dilatation and impact of shunt volume and increased work load on cardiac function has not been quantified and remains unclear.

Echocardiography is currently used to carry out neonatal cardiac assessment. Several echocardiography studies have shown an increase in left ventricular cavity dimension and stroke volume in PDA infants [11],[13], but have been limited by the accuracy of echocardiography to quantify intra cardiac cavity dimensions and flow volumes [14],[15]; measurements have been flawed by significant observer variability and poor agreement to reference methods [16]. Furthermore none of these studies have been able to associate cardiac function with ductal shunt volume and resultant systemic flow. Consequently robust normative ranges in the preterm population are scarce.

Balanced steady state free precession (SSFP) has become a prominent diagnostic and functional tool in cardiovascular magnetic resonance (CMR), due to the excellent blood-myocardium contrast from the inherent T2/T1 weighted contrast and very high signal to noise efficiency [17]. CMR provides accurate and reproducible cardiac dimension, function [18] and LVO measures in adults [19],[20] and in cardiac failure patients [21]. A previous preliminary study demonstrated that functional assessment with CMR in neonates was feasible [22], however images were acquired without the use of specialist receive coils, such that image resolution was limited.

The aim of this study was to firstly quantify and validate left ventricular (LV) dimension and function parameters in preterm infants using an optimized SSFP stack sequences [23]. Secondly, to establish normative ranges in the preterm and term population. Thirdly, to carry out a preliminary observational study to investigate the impact of PDA on LV dimension and function.

## Methods

The study was approved by the North West London Research Ethics Committee (06/Q0406/137) and written informed parental consent was obtained in all cases. All echo and CMR scans were carried out for research purposes only.

### Study cohort

CMR was performed on 45 infants who were inpatients at St Thomas’ and Queen Charlotte’s and Chelsea Hospitals. All infants had an echo performed within 24 hours of the CMR scan by an operator with >10 years of experience (AMG) in neonatal echocardiography. Due to the lack of consensus on optimal PDA management, treatment approach likely reflects the approach of the clinical team rather than the significance of the duct [24]. The Queen Charlotte’s and Chelsea Hospital neonatal unit has adopted an approach of non-intervention for the PDA, leaving the duct to close naturally in most cases; providing the opportunity to study the duct at a point in its natural history. All echocardiographic images were acquired using a Vivid 7 ultrasound machine (GE Healthcare, Milwaukee, WI) with a 10 MHz sector probe. Colour Doppler echo assessment was used to determine the absence or presence of a PDA. PDA shunt volumes were determined by phase contrast (PC) CMR analysis [12]. Of these 45 infants, 1 was born a term infant.

Twenty nine neonates (controls) median (range) gestational age (GA) at birth 33^+2^(25^+5^ – 39^+1^) weeks, corrected GA at scan (cGA) 34^+6^(31^+1^-39^+3^) weeks, birth weight (BW) 1820(695 – 3045) grams and current weight at scan (CW) 1880(790 – 3045) grams had closure of the ductus arteriosus confirmed by echo. Sixteen infants had PDA determined by echo, median (range) GA 26^+5^(24^+3^ – 34) weeks, cGA 30^+2^(27^+3^ – 36^+1^) weeks, BW 955(525 – 2000) grams and CW 1130(660 – 2400) grams.

PDA was treated in 2 infants, both with Ibuprofen 2 days and 59 days prior to the scan. Both were still patent at the time of the CMR scan. None of the PDA infants subsequently underwent surgery to close the duct. All of the 16 PDA infants survived and there were no incidences of necrotising enterocolitis in this cohort. No infant included in this study went on to receive further treatment for prolonged ductal patency.

### CMR acquisition

All infants were scanned using acoustic ear protection, pulse oximetry, vector ECG monitoring and without sedation or anesthesia, as described previously in Merchant *et al*. [25]. Axillary temperature was monitored throughout the scan for all individuals and remained stable in all cases. Images were acquired free breathing, as previously demonstrated the nature of neonatal respiration is predominately shallow but rapid, minimizing the bulk motion of the heart within the chest cavity [23]. Seventeen infants required low flow supplemental oxygen or nasal continuous positive airway pressure via an MR compatible system, but all infants were stable and tolerating full enteral feeds during the scan. None of the infants were mechanically ventilated during the scan.

Due to the high heart rates, lack of patient cooperation and subject size performing successful CMR in the neonatal population requires a tailored approach. Therefore scanner and parameter optimization fully described in Price *et al*. [23] were utilized in this study. This study showed good agreement between LVO and SV measures from stack and PC CMR data [23]. Data was acquired on a Philips 3-Tesla MR Achieva scanner (Best, Netherlands) using a specialised 8 channel pediatric body receive coil for infants > 2 kg and an 8 channel small extremity receive coil for infants < 2 kg.

LVO was quantified in all but 6 infants due to patient unrest from PC CMR. PDA shunt volume was indirectly calculated from 3 PC sequences quantifying LVO, upper and lower body flow acquired during the same CMR scan (acquired in-plane resolution = 0.6 × 0.6 mm, slice thickness = 4 mm, TR/TE = 5.9/3.1 ms, velocity encoding ranged from ±60-150 cms^−1^ depending on slice position), full details of the PC CMR sequences are given in Broadhouse *et al.*[12]. Ductal shunt volume was calculated in all but 3 PDA infants as these infants became unsettled.

A gated 2D SSFP short axis 10 slice stack optimized for neonatal CMR [23] (acquired in-plane resolution = 1 × 1 mm, slice thickness = 4 mm, TR/TE = 3.8/1.9 ms, flip angle = 35°) was placed over the heart, aligned with the mitral valve using previously acquired pilot scans. Negative slice gap was adjusted for individual infants so that full coverage from apex to base of the left ventricle was achieved. Number of signal averages (NSA) ranged between 2 and 8 depending on size and stability of the infant. No acceleration methods were used in this study; acquisition time ranged between 2 and 8 minutes.

### Data processing

Quantification of LVO and ductal shunt volume from PC sequences [12] was performed using a commercial workstation (Philips ViewForum). Automated vessel edge detection was used for all vessels of interest, with manual correction where necessary. Stacks were segmented and LV function quantified using freely available software Segment v1.8 R1172 (http://segment.heiberg.se) [26]. Myocardial borders were defined by performing a detailed manual tracing of the epi and endocardium. Long axis motion (LAC) was corrected for by measuring the mitral valve displacement on a 4 chamber sequence between the end diastolic (ED) and end systolic (ES) time points taking an average of three measures.

Myocardial and ventricular cavity volumes were determined by summing the area of each slice over the length for ED and ES time points, the two volumes were then averaged. Myocardial mass was calculated by multiplying the left ventricular myocardial volume by 1.05 g/ml [27].

Myocardial wall thickness and fractional thickening assessment was carried out using the 6 mid cavity sections as defined by the 17 sector American Heart Association model [28]. Papillary muscles and trabeculae were excluded from the blood pool area for evaluation of the blood volume used to calculate end diastolic volume (EDV), stroke volume (SV), LVO, and ejection fraction (EF). Papillary muscles and trabeculae were included in the blood pool volume for calculation of the myocardial wall parameters: wall thickness, fractional thickening, and left ventricular (LV) mass.

### Statistical analysis

Intra- and inter-observer variability analysis of EDV, ESV, SV, LVO, EF, LV mass and LAC was carried out in 10 infants picked at random from the control cohort. Bland-Altman analysis was used to assess agreement between the 2 measures. Mean difference of measures, limits of agreement (LOA) and repeatability index (LOA/mean of measures, RI) were also calculated.

The LV output measures LVO, SV, EF and ventricular dimensions EDV and LV mass normalized by weight at scan (as is convention in the neonatal population) were plotted against corrected gestational age to visually assess the impact of PDA on LV measures. EDV was plotted against SV to investigate the relationship between filling and stroke volume in control and PDA infants to look for evidence of alteration in the Frank Starling curve [29]. LV mass was plotted against EF to investigate the relationship between the apparent enlarged left ventricle and cardiac function in PDA infants.

La Place’s law states a linear relationship between ventricular diameter and wall stress. Therefore to maintain cardiac function, any scenario producing increased left ventricular diameter would be expected to also produce a proportional increase in myocardial thickness. To examine whether any increase in LVmass in infants with PDA is proportionate or disproportionate to extent of LV blood volume change, the ratio between LV myocardial volume and LV blood pool volume at end diastole (LVmass/EDV, where in this case LVmass is = LVmass/1.05 g/ml, the volume of myocardium) was examined.

An unpaired Student’s t-test compared EDV, SV, LVO, EF, LVmass, ED wall thickness, fractional thickening, and LVmass/EDV ratio in infants with and without PDA. Linear regressions were carried out in control infants to determine association between EF and the ventricular measures: LVmass and EDV. It was recognised that by the nature of PDA the PDA and control infants were different cohorts with respect to GA, cGA and postnatal age. Consequently multiple linear regressions were carried out in solely PDA infants to determine association between LV mass, EDV and LVmass/EDV ratio with ductal shunt volume in these infants correcting for cGA and postnatal age. Association between EF and LVmass, EDV and ductal shunt volume was also investigated in the PDA infants.

To assess whether differences in LVmass could be related primarily to illness severity rather than PDA shunting, the association between number of days on respiratory support from birth to discharge and LVmass correcting for cGA, postnatal age and ductal patency was investigated. Statistical significance was determined by a p value < 0.05.

## Results

**Figure 2:**
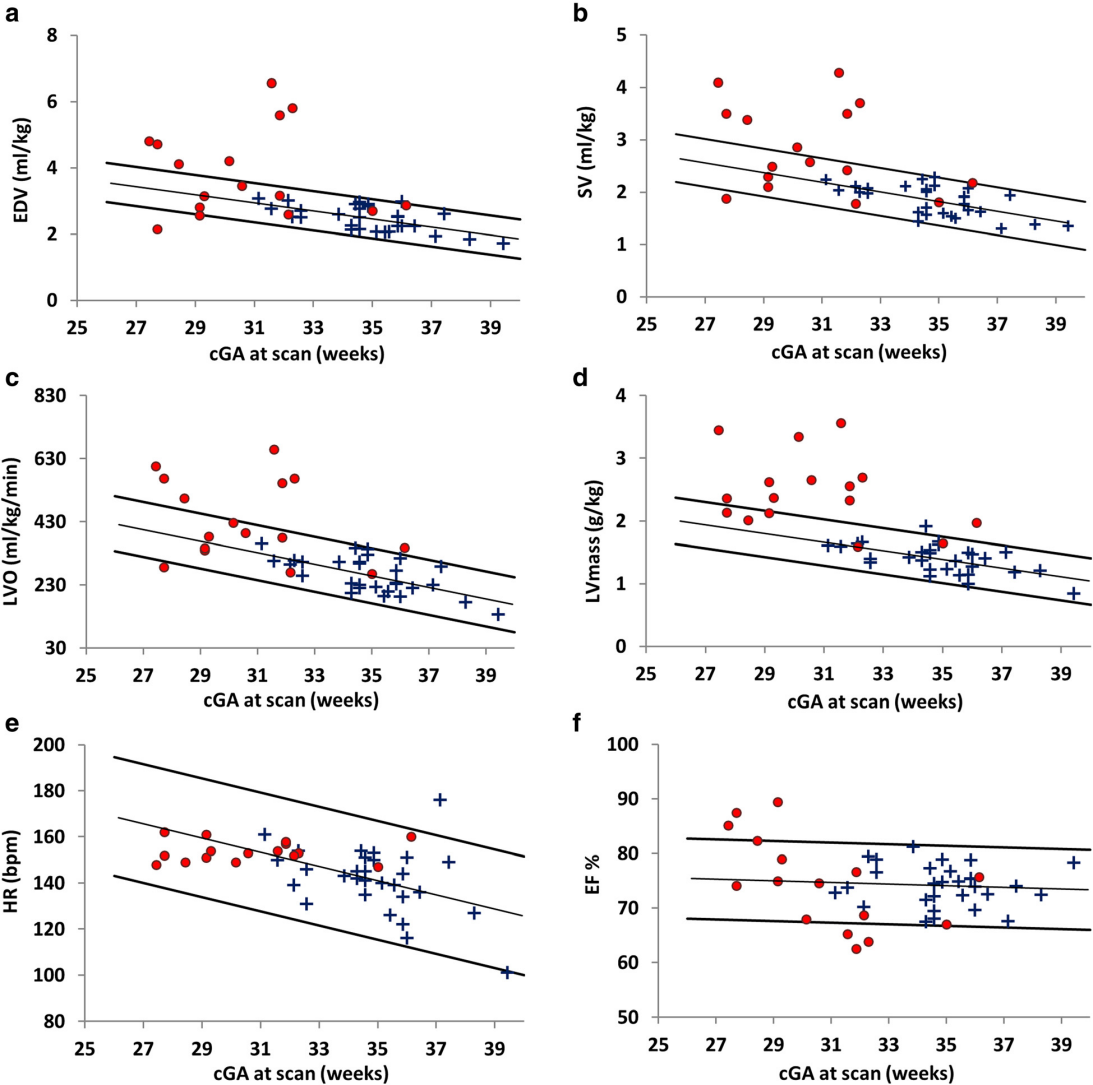
Normative ranges with trend line and 95% confidence limits of a: end diastolic volume (EDV), b: stroke volume (SV), c: left ventricular output (LVO), d: left ventricular (LV) mass, e: heart rate (HR) and f: ejection fraction (EF) in 45 infants with (●) and without (+) PDA.

10 control infants with median (range) GA 33^+2^(26^+2^ – 35^+4^) weeks, cGA 35^+2^(32^+1^ – 37^+3^) weeks, BW 1860(1040 – 2770) grams and CW 1980(1060 – 2770) grams were assessed for intra- and inter-observer variability analysis. Bland-Altman intra- and inter-observer variability analysis for the LV dimension and functional parameters showed good agreement between all LV dimension and functional parameters, RI ranged between 4.2 – 13.9% for intra-observer variability and 6.6 - 23.1% for inter-observer variability (Table 1). Mean and range of LV volume and function parameters from control infants are shown in Table 2. Normative ranges (Figure 2a, b, c, d, e and f) show a decrease in EDV, SV, LVO, LV mass (normalized by weight at scan) and EF with increasing cGA.

**Table 1:** Bland-Altman intra- and inter-observer analysis for EDV, ESV, SV, LVO, EF, LVmass and LAC in 10 control infants

		**Intra-observer**			**Inter-observer**	
	**Mean diff %**	**RI %**	**LOA**	**Mean diff %**	**RI %**	**LOA**
**EDV ml**	3.6	7.1	−0.2 – 0.5	−0.5	11.6	−0.4 – 0.6
**ESV ml**	9.2	13.6	−0.1 – 0.3	8.3	19.0	−0.1 – 0.4
**SV ml/min**	1.6	8.7	−0.3 – 0.4	−3.5	12.7	−0.6 – 0.4
**LVO ml/min**	1.8	8.9	−38.3 – 56.9	−3.6	12.5	−87.7 – 48.8
**EF %**	−1.9	4.2	−4.6 – 1.7	−3.2	6.6	−7.3 – 2.5
**LVmass g**	9.2	13.9	−0.1 – 0.7	18.1	16.2	0.1 – 1.0
**LAC mm**	−3.4	12.7	−0.9 – 0.5	−11.11	23.1	−2.2 – 1.0

**Table 2:** Mean and range of LVO, SV, EDV, LVmass (normalized by weight at scan), EF and HR in control infants

	**Mean**	**Range**
**LVO ml/kg/min**	260	137 - 360
**SV ml/kg**	1.83	1.31 - 2.29
**EDV ml/kg**	2.47	1.73 - 2.29
**LVmass g/kg**	1.39	0.85 - 1.91
**EF %**	74	68 - 81
**HR bpm**	141	101 - 176

Of the 16 PDA infants (13 assessed by PC CMR), the median (range) ductal shunt volume was 26(3.4-74.2)% of LVO and 86.0(17.0 – 434.8) ml/kg/min. Table 3 shows ductal shunt volume as a % of LVO and in ml/kg/min for all 13 infants. Unpaired Student’s t-tests showed that EDV, SV, LVO, ED wall thickness, LVmass and LVmass/EDV are significantly increased in PDA infants. There was no significant difference between EF and fractional thickening in both groups (Table 4). For visual analysis EDV, SV, LVO, LVmass (normalized by weight at scan), HR and EF were plotted against cGA (Figure 2a, b, c, d, e and f). Seven of the 16 PDA infants have EDV, SV and LVO above the population 95% confidence limits. One PDA infant has EDV and SV below the population 95% confidence limits. LVmass is dramatically increased in PDA infants with all but 5 above the population 95% confidence limits. Heart rate in all PDA infants remained within the population 95% confidence limits. Mean (range) EF was found to be 74(68 – 81)% in control infants. All but 3 infants had an EF within or above the population 95% confidence limits and mean (range) was found to be 75(63 – 89)%. All PDA infants had EF above the threshold of >50% for normal EF in adults [30] and had mean EF above the mean EF of 69% found in healthy pediatric subjects aged between 8–12 years [31].

**Table 3:** Ductal shunt volumes: GA, cGA, birth weight (BW), weight (Wt) at scan and corresponding ductal shunt values as determined by PC CMR for 13 infants with PDA

**GA weeks**	**Cga weeks**	**BW kg**	**CW kg**	**Shunt volume % LVO**	**Shunt volume ml/kg/min**
24^+3^	27^+3^	0.53	0.66	74.2	435
26^+3^	28^+3^	0.95	0.95	66.5	336
28^+4^	31^+4^	1.40	1.43	62.0	359
25^+4^	31^+6^	0.68	1.13	52.7	275
26^+1^	30^+1^	0.99	1.08	51.6	221
27	29^+2^	1.14	1.14	48.7	169
27	29^+1^	1.18	1.18	26.0	86
26^+2^	36^+1^	0.91	2.40	23.5	72
25^+5^	27^+5^	0.81	0.81	15.5	45
25^+3^	30^+4^	0.85	0.88	15.0	58
27	29^+1^	0.89	0.89	13.5	46
27^+2^	31^+6^	0.90	1.22	12.1	39
31^+3^	32^+1^	1.39	1.39	3.4	17

**Table 4:** t-test analysis of left ventricular output, function and hypertrophic measures showing mean and p-values for control and PDA groups

**T-tests**			
	**Mean controls**	**Mean PDA**	**p value**
**LVO ml/kg/min**	260(56)	432(127)	<0.001
**SV ml/kg**	1.82(0.29)	2.81(0.83)	<0.001
**EDV ml/kg**	2.47(0.38)	3.83(1.34)	0.001
**LV mass g/kg**	1.39(0.23)	2.46(0.59)	<0.001
**ED Myo thickness mm/kg**	1.07	2.07	<0.001
**LVmass/EDV**	0.54(0.08)	0.68(0.17)	0.02
**EF %**	74(4)	75(8)	0.80
**Fractional Thickening %**	77	80	0.69

**Figure 3:**
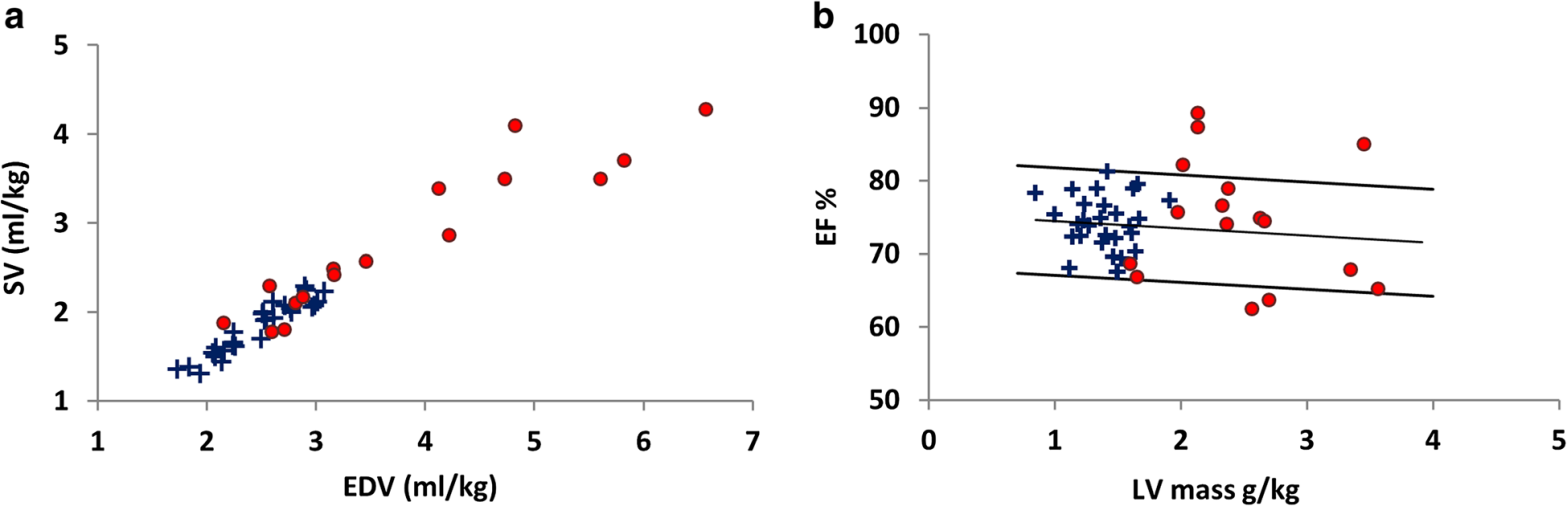
**Analysis of left ventricular mass. a**: Population Frank Starling curve and **b**: left ventricular (LV) mass plotted against ejection fraction (EF) for infants with (●) and without (+) PDA.

Plotting a population Frank Starling curve (EDV against SV) revealed a linear trend irrespective of ductal patency (Figure 3a). All but 2 PDA infants had EF within or above the 95% confidence limits when plotted against LV mass (Figure 3b).

**Figure 4:**
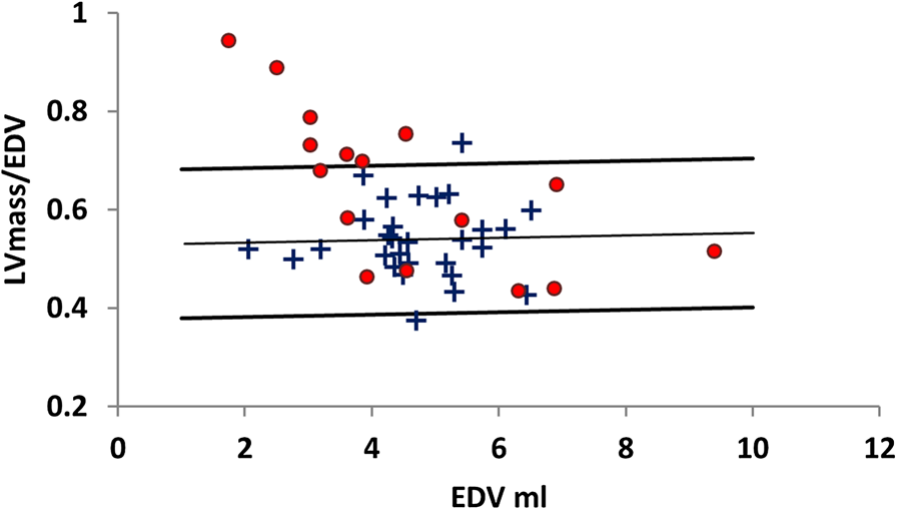
** Normative range of the myocardial volume to end diastolic volume (LVmass/EDV) ratio against EDV in infants with (●) and without (+) PDA.** The relationship between increased LVmass/EDV ratio and decreased EDV is illustrated.

The ratio of LVmass/EDV is approximately constant with increasing EDV in control infants. An inverse relationship is observed in the PDA infants where LVmass/EDV decreases with increased EDV and with 7 of the PDA infants above the 95% confidence limits. Figure 4 highlights the inverse relationship between EDV and LVmass/EDV ratio.

Linear regression analysis showed that there was no significant relationship between EF and EDV or LVmass in control infants. In PDA infants there was no significant association between EF and ductal shunt volume or LV mass, however a significant inverse relationship was found between EF and EDV (p = 0.02).

Multiple linear regression analysis showed a significant positive association between LV mass and ductal shunt volume, and between EDV and ductal shunt volume both persisted when correcting for cGA and postnatal age in multiple linear regression (both with p < 0.01). A significant inverse relationship between LVmass/EDV and ductal shunt volume persisted when correcting for both cGA and postnatal age (p < 0.01). No significant association between number of days on respiratory support and LV mass correcting for cGA, postnatal age and ductal shunt volume was found.

## Discussion

The preterm cardiovascular system, the cardiac function and the associated pathologies are poorly understood. Although functional CMR is well established in adult and pediatric populations the translation to the neonatal cohort has been challenging due to the nature of the subjects. This and the limitations in echocardiography have meant that robust neonatal normative ranges are scarce. The recent application of CMR in neonates [12],[22],[23] has made it possible to evaluate cardiac dimension and function in this cohort. In this study volumetric and functional CMR assessments with the optimized acquisitions [23] have been quantified and validated. Preterm normative ranges of left ventricular dimension and function were determined and utilized for a preliminary investigation of the impact of PDA.

Bland-Altman analysis of intra- and inter-observer variability indicates good agreement between EDV, SV, LVO and EF measures (RI ranged between 4.2 -12.8%, Table 1). Intra- and inter-observer LV mass and ESV measures showed reasonable agreement (RI = 13.6, 13.0 and 19.0, 16.2% respectively). This reduced repeatability in ESV is due to the variability in LAC measures (RI = 12.7 and 23.1%). Post processing software corrected for long axis motion in integer values. This is not problematic in adults but can be significant in infants with basal to apex lengths of ~ 25 mm. An increment of 1 mm could lead to an alteration in EF of 2% and therefore even larger difference in ESV. These repeatability measures were a marked improvement over echo values [14]–[16] and are comparable to pediatric CMR studies [31]. Robbers-Visser *et al.*[31] found a coefficient of variance ranging from 2.1 – 13.9% for intra- and inter-observer variability, this would imply an RI of 4.1 – 27.2% (intra- and inter-observer variability RI range = 4.2 – 23% in this study).

The quantitative measures (EDV, SV, LVO, LVmass and HR) showed a linear decrease with increasing cGA (Figure 2a, b, c, d, and e). For the range of cGA of this cohort the increase in weight is predominantly attributed to an increase in adipose tissue which requires proportionally less blood flow than organs, hence a decrease in LV output and dimension when normalized by weight at scan is expected with increasing cGA. EF remained relatively constant across cGA with a mean (range) EF of 74(68 – 81)%. For comparison a previous CMR study assessing normal biventricular function, volumes and mass in a pediatric cohort (8-12 yrs) showed an average EF of 69% [31]. Linear regression analysis also showed no significant relationship between EF and GA, cGA, EDV or LV mass in control infants, indicating that these infant’s hearts are well within their comfortable functioning range.

Visual assessment from normative ranges and unpaired t-tests showed that in PDA infants EDV was significantly increased, suggesting volume loading on the heart. In addition SV, LVO and LVmass were increased, indicating that these hearts are experiencing an increased workload. Despite the widely held clinical belief that PDA presents with increased HR, a sign of distress in neonates, even in the presence of large ductal shunt volumes (up to 74% of LVO) PDA infants had HR consistently within the normal range. This indicated that LVO was increased in PDA infants primarily by increased SV due to a larger EDV than increase in HR. Despite the increase in LV volume loading and workload there was no significant difference in EF between control and PDA infants. The data in this study supports the idea that an individualized treatment approach is needed and may suggest that not all large shunt volumes lead to heart failure.

This preliminary data presented here may suggest an overall maintenance of contractile function in PDA infants. All PDA infants in this study had EF well above the threshold defining normal EF, (>50%) mean (range) 75(63 – 89)% [30] and were within range of pediatric data (mean EF = 69%) [29]. From the Frank Starling curve all PDA infants appear to remain on the same linear trend as control infants; there is no evidence of a drop in stroke volume with very large EDV. Indicating that in general the Frank Starling mechanism adequately compensates for the mechanical disadvantage imposed by Laplace's law (the linear relationship between ventricular diameter and wall stress) in these enlarged hearts. While a significant inverse relationship was found between EF and EDV (p = 0.006) in PDA infants, all but 3 infants had EF within or above the normal range.

While contractile function seems to be generally maintained in PDA infants there is clearly a significant increase in LVmass. This increase is not explained by differences in cGA and postnatal age. Assessment of the correlation between ductal shunt volume and this enlargement was investigated by considering the relationship between EDV and the ratio of LVmass/EDV. This ratio was almost constant in control infants. The LVmass/EDV was higher overall in PDA infants. However, there was a significant association between increased LVmass/EDV and decreased ductal shunt volume in PDA infants. Indicating that infants with lower shunt volumes may have hypertrophic growth which is more disproportionate than infants with high shunt volumes.

This relationship appears counter intuitive and warrants examination in a larger cohort of infants. However, lower ductal shunt volumes could be indicative of high pulmonary vascular resistance, which could lead to increased right ventricular afterload and subsequently lead to overall increased myocardial mass in the lower shunt volume infants. Although, there was no significant association between number of days on respiratory support from birth to discharge and LVmass correcting for cGA, postnatal age and ductal shunt volume, this could be due to the small number of infants and the number of confounding factors.

### Study limitations

This study has three significant limitations. Firstly due to the nature of PDA (increased incidence that is inversely related to gestational age) there was a significant difference in cGA between control and PDA infants: there are relatively few infants <30 week cGA without a PDA in this study. Graphic comparison between control and PDA infants was performed by linear extrapolation of the 95% confidence limits, it is feasible that infants <30 weeks cGA without a PDA do not follow this linear trend. The significant differences from t-test analysis in LV volumetric data between the control and PDA groups could be due to the difference in GA, cGA and postnatal age of the 2 groups and not the presence of a PDA. The resultant impact of the immature circulatory system are numerous, complex and poorly understood as both the body tissues and the circulation and heart supplying them are immature. Many studies are now suggesting that the association between pathologies and PDA could be casual rather than causal and this may also be the case with the LV volumetric data presented in this study.

Secondly a relatively small number of PDA infants were studied at a time of clinical stability and outside the transitional period. We have presented data suggesting that cardiac function is maintained even in infants with a high ductal shunt volume. However, it may be that at other points, during a time of clinical instability and particularly in the first few days of life during the circulatory transition where dramatic changes occur in preload conditions, myocardial contractility and systemic and pulmonary vascular resistance in PDA infants may not be within this regime.

Thirdly we have presented data at only one time point; we have no temporal data of the ductal shunt volume and resultant cardiac status. The duration of the heart’s transition to adapt to the increased volume load or whether this enlargement persists long after the closure of the duct is unknown. Although there was no significant association between EF and shunt volume the ventricular dimensions were significantly increased, whether this increase in LV mass is due to hypertrophy or hyperplasia is difficult to determine and if this is evidence of pathological ventricular remodeling is unclear.

## Conclusions

In summary, this study presents data validating CMR assessment of LV volumes and function in preterm and term infants, a cohort in which data is scarce. Normative ranges were established in this population and then utilized to investigate left ventricular dimension and function in the presence of a PDA. LVO, EDV and LV mass were found to be significantly increased in PDA infants. Moreover a significant association was found between increased LV mass and ductal shunt volume. A significant increase in LVmass/EDV was found in PDA infants suggesting that these hearts may be disproportionately hypertrophic. However, EF and fractional thickening remained within the normal range and no association was found between EF and ductal shunt volume. In addition, none of the PDA infants appear beyond the peak of the Frank Starling curve. This may suggest that function is generally maintained in these enlarged hearts. The data highlights the fact that more study is needed to aid individual treatment approach.

## Abbreviations

BW: Birth weight

cGA: Corrected gestational age

CW: Current weight at scan

ED: End diastolic

EDV: End diastolic volume

EF: Ejection fraction

ES: End systolic

GA: Gestational age

LAC: Long axis motion correction

LOA: Limits of agreement

LV: Left ventricular

LVO: Left ventricular output

NSA: Number of signal averages

PC: Phase contrast

PDA: Patent ductus arteriosus

RI: Repeatability index

SSFP: Steady state free precession

SV: Stroke volume

## Competing interests

There are no other conflicts of interest.

## Authors’ contributions

KMB participated in the design of the study, acquisition and data collection, performed the data and statistical analysis and drafted the manuscript. AEF participated in data acquisition. ANP participated in the design of the study, sequence acquisition and aided in drafting the manuscript. GD participated in sequence acquisition. DJC participated in the data analysis. ADE participated in the study design and helped draft the manuscript. JVH participated in the study design and aided with the statistical analysis. AMG conceived the study, participated in the study design and aided with the draft of the manuscript. All authors read and approved the final manuscript.

## Acknowledgements

Supported by the Garfield Weston Foundation, the Medical Research Council, the National Institutes for Health Research Imperial College Comprehensive Biomedical Research Centre and the National Institute for Health Research (NIHR) Biomedical Research Centre at Guy's and St Thomas’ NHS Foundation Trust and King's College London. ANP and AMG are supported by an MRC Clinician Scientist Fellowship awarded to AMG. Study sponsors had no involvement in data collection, analysis or interpretation.
